# Non‐Palpable Contraceptive Implant Evaluation and Removal: A Protocol That Can Be Adopted for Practice in Low‐Middle Income Countries

**DOI:** 10.1002/puh2.70086

**Published:** 2025-08-01

**Authors:** Abraham Fessehaye Sium, Amani Nureddin Abdu, Jaclyn M. Grentzer, Matthew F. Reeves, Sarah Prager

**Affiliations:** ^1^ Department of Obstetrics and Gynecology St. Paul's Hospital Millennium Medical College Addis Ababa Ethiopia; ^2^ Department of Population, Family, and Reproductive Health Johns Hopkins Bloomberg School of Public Health Baltimore Maryland USA; ^3^ Department of Obstetrics and Gynecology George Washington University School of Medicine and Health Sciences Washington District of Columbia USA; ^4^ DuPont Clinic Washington District of Columbia USA; ^5^ Department of Obstetrics and Gynecology, Division of Complex Family Planning UW Medicine Seattle Washington USA

**Keywords:** deep contraceptive implant | deep implant | non‐palpable implant | non‐palpable implant removal

## Abstract

Currently, there are four implants that a family planning provider might encounter for removal: Jadelle (two radiopaque, flexible cylindrical implants, 43 mm × 2.5 mm, consisting of dimethylsiloxane/methylvinylsiloxane copolymer core enclosed in thin‐walled silicone casing. Each implant contains 75 mg of levonorgestrel, FDA approved for 5 years of use); Levoplant (which is very similar to Jadelle but is only FDA approved for 4 years); Nexplanon (a single, radiopaque rod (40 mm × 2 mm) made of ethylene vinyl acetate copolymer. Each implant contains 68 mg etonogestrel. FDA approved for 3 years of use); and Implanon (a single, non‐radiopaque rod (40 mm × 2 mm) made of ethylene vinyl acetate copolymer. Each implant contains 68 mg etonogestrel. FDA approved for 3 years of use). Most implants can be easily palpated after insertion as well as at the time of removal, but in a small number of cases, the implant may not be palpated due to a variety of factors: a clinician inserted the implant deeper than the subcutaneous layer or in a place other than is recommended; the implant was not inserted at all; a woman has gained significant weightand there is increased subcutaneous fat overlying the implant; or the implant has migrated away from the insertion site. Of importance is that if the implant cannot be palpated, no attempt should be made to remove it until it can be localized. Given the rising use of long‐acting reversible contraceptives (LARCs), a standardized approach to difficult implant removals is essential, especially in low‐middle income countries (LMICs). In this protocol, we demonstrate a step‐by‐step approach for identification and removal of non‐palpable implants that can be adopted for practice across LMICs. This protocol may also serve as a foundation for broader clinical guidelines and training programs.

## Graphical abstract

This protocol provides a comprehensive stepwise approach for localization and removal of non‐palpable contraceptive implants that can be adopted for practice in low‐middle income countries such as Africa. In general, most non‐palpable contraceptive implants can be localized with ultrasound. Hence, no attempt should be made for a blind remove.

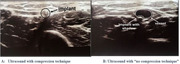

## 1 Introduction

Deep implant insertions are estimated to occur approximately one out of every 1000 insertions, which is similar to the perforation rate of intrauterine devices [1, 2]. Severe complications associated with deep placement, such as intravascular placement with pulmonary embolization, are estimated to occur in just over one patient per 1 million implants sold [3].

When a deep/non‐palpable implant is diagnosed, referral to a specialty center with physicians who have expertise localizing and removing non‐palpable implants is essential [4]. Instructions for subdermal implant insertion have changed over the past decade, primarily inspired by rare complications related to deep insertions, including intravascular placement and pulmonary artery migration [4, 5, 6, 7]. Initially, the manufacturer suggested insertion into the sulcus between biceps and triceps muscle. The most recent recommendation for optimal insertion is between the dermis and subcutaneous tissue, approximately 8–10 cm proximal to the medial epicondyle of the humerus and 3–5 cm posterior to the sulcus between the biceps and triceps muscles, with the intent to minimize the likelihood of neurovascular injury should deep insertion inadvertently occur [3, 8]. This location intends to avoid the large blood vessels and nerves lying within and surrounding the sulcus. When palpable at the anticipated location, the implant is usually easy to extract, whereas deeply placed implants are frequently nonpalpable and difficult to locate [9].

Attempts to blindly remove an implant could lead to injury of nearby vessels and nerves [4, 10]. Ultrasound is the most widely used technique [11, 12], but the addition of barium sulfate also allows x‐ray radiography as a first option for localization. Due to the low prevalence (1:1000) of non‐palpable implants, no standardized extraction method is available [8]. Health care providers have described various advanced techniques to localize and remove non‐palpable implants [4, 13, 14, 15, 16, 17, 18, 19]; however, the reports typically only include descriptions with a few patients and often involve costly resources such as interventional radiology, fluoroscopy, and removal in the operating room. Previously published techniques for removal of six‐rod implant systems include the Emory method, the pop‐out method, the “U”‐technique, the hook‐traction method, the needle‐elevation method, or the modified needle‐elevation method [20, 21, 22, 23, 24, 25].

A literature search on management of non‐palpable single‐rod implants has revealed different approaches, with some describing extraction under general anesthesia [9]. Others have described removal with real‐time ultrasonography guidance [26] or invasive US‐guided localization, whereby a hook wire marker is placed near to the implant under US guidance [27]. Chen et al. from California [28] presented the use of a modified vasectomy clamp for extraction. Recently, in 2021, El‐Hadad et al. from Switzerland demonstrated removal of non‐palpable etonogestrel implants after fixation with a curved needle [29]. Here, we present a different and more comprehensive protocol, with a step‐by‐step approach from evaluation of non‐palpable implant through removal, to be used to easily localize and remove deep contraceptive implants. Undetected and improperly removed implants could compromise contraceptive efficacy and client trust. This protocol could help reduce such risks and improve service delivery quality in low‐middle income countries.

## 2 Implant Localization

### 2.1 Palpation

Ask the following questions prior to attempting to palpate the implant:

- When was your implant inserted?
- Into which arm was it inserted?
- Is this your first implant? If no, can you point out your various insertion and removal scars?
- Can you identify the insertion scar for THIS implant?
- Has anyone tried to remove this implant already?
- Have you ever been able to feel this implant?

Steps for palpation

- Palpate starting at the insertion site scar.
- Palpate the entire upper arm starting from the level of the epicondyle to the axilla, focusing on the area overlying and adjacent to the groove between the biceps and the triceps, and the entire axilla.
- If still not palpated, palpate the entire upper arm.
- If still not palpated, evaluate the contralateral upper arm in the same systematic fashion.
- If still not palpated, or if unsure if what you felt was the implant, proceed with imaging.

### 2.2 Imaging

In general, a distinction between radiopaque (e.g., Nexplanon) and non‐radiopaque (e.g., Implanon) implants is clinically important for localization using imaging modalities. This difference underlines the necessity of choosing appropriate diagnostic tools (e.g., ultrasound vs. x‐ray or MRI) based on implant type

#### 2.2.1 Ultrasound

##### 2.2.1.1 Overview

- All implants can be visualized on ultrasound.
- Must use high‐frequency linear array transducer (12–15 MHz).
- To enhance implant visibility if using lower frequency ultrasound transducer, you can scan through an IV fluid bag or glove filled with water overlying the area of the suspected implant location.

##### 2.2.1.2 Procedure steps

**FIGURE 1:**
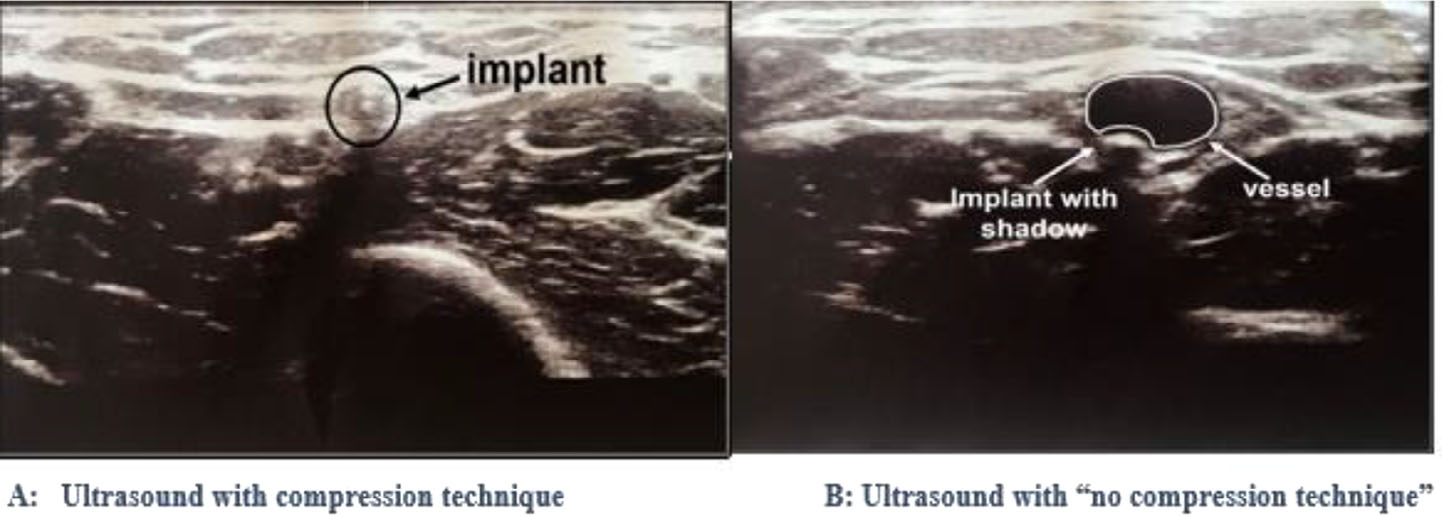
Ultrasound findings of Nexplanon with (A) “compression” and (B) “no compression” technique [30].

1. Position the patient to be lying flat on her back with her arm bent into a goalpost position.
2. Adjust the depth of your probe to ensure that you are not including the humerus.
3. Set the focus to be 0.5–1 cm deep.
4. Start with the transducer in the transverse position and scan from the presumed insertion site proximally towards the axilla.
5. If not initially visualized, broaden your scan area to include the entire upper arm from epicondyle to axilla.
6. If visualized:
  - Measure the depth of both the proximal and distal ends in relationship to the skin and to the fascia muscularis. Ensure that you are not compressing the tissue by using the “no compression” sonographic technique (Figure 1). They allow you to have accurate implant depth measurement as well as avoid compression of blood vessels that can be easily detected with this technique.
  - Turn the transducer 90 degrees to obtain longitudinal view of the implant and measure the implant's length to confirm it is, in fact, the implant.

##### 2.2.1.3 If implant is NOT seen on ultrasound on one arm, scan the other arm. If it still cannot be seen, obtain upper arm x‐ray

#### 2.2.2 X‐ray

##### 2.2.2.1 Overview

   

- Nexplanon contains barium sulfate and is radiopaque.
- Jadelle rods do not contain barium but are still radiopaque.
- Implanon is NOT radiopaque and cannot be visualized on x‐ray.

##### 2.2.2.2 Procedure details

**FIGURE 2:**
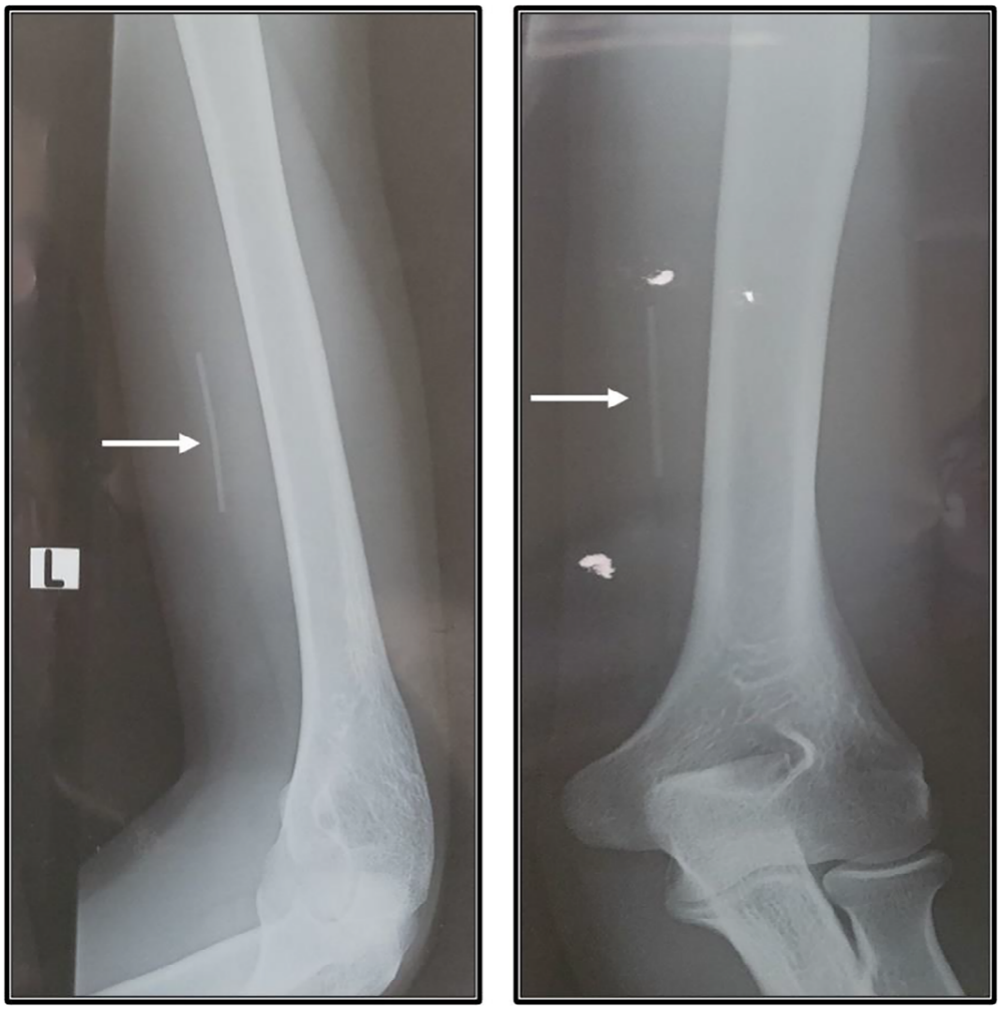
Lateral and posteroanterior x‐ray of the left arm (arrow indicates the implant‐Nexplanon).

1. Must request upper arm x‐ray with BOTH anteroposterior (AP) and lateral views (Figure 2).
2. For Jadelle, request x‐ray to be set at 50–55 kV and 4–5 mA with an exposure time of 0.03 s.
3. Review all images for yourself—regardless of the radiology report.

##### 2.2.2.3 If not seen on upper arm x‐ray

**FIGURE 3:**
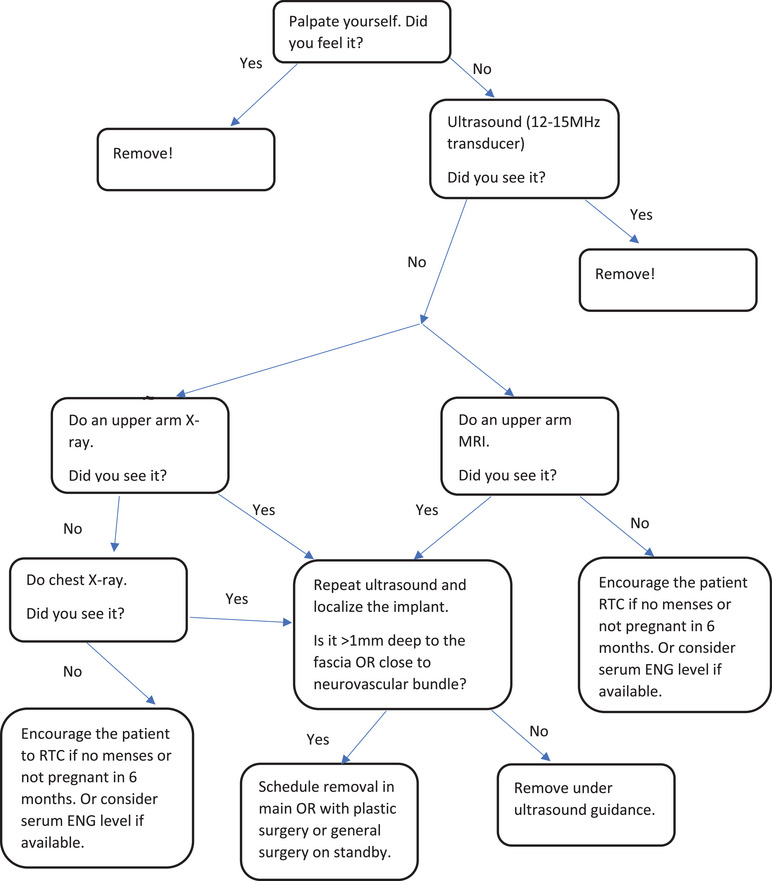
Non‐palpable implant localization and removal algorithm *(N.B: If implant is seen in the pulmonary vasculature on X‐ray or MRI, a watch ‐and‐ wait approach or removing it by interventional radiologist should be considered after discussing the benefits and risks of retrieving the implant with the patient and depending on whether it is complicated by embolization or not)*.

1. Obtain chest x‐ray (CXR), AP, and lateral views.
  - If not seen on CXR, but you are confident that this is a Jadelle or Nexplanon, then assume the implant was never placed (Figure 3). If resources allow, consider MRI (if patients can afford it and wish to have more diagnostic certainty). If the patient is attempting pregnancy, have her return in 6 months if her menses does not return OR if she is not pregnant by then.
  - If not seen on CXR, and you suspect that it is an Implanon, then obtain MRI that includes chest and bilateral upper arms.

#### 2.2.3 MRI

- Very rarely, localization of Implanon when it cannot be seen on ultrasound may be made with MRI in settings where MRI is available. If Jadelle or Nexplanon cannot be identified on chest x‐ray, MRI may be considered after discussion of facility and patient resources.
- MRI protocol should include T1 sequence, sagittal and coronal planes, without fat suppression, and in slices ≤2 mm.
  - Implant is easier to confidently identify on the T1 sequence as differentiation from subcutaneous veins is more marked.
  - Sagittal and coronal planes should be the initial choice given that implants are usually inserted parallel to the humerus.
  - Implant will be hypointense on all sequences because it does not contain free water or free protons.
  - Do not use fat suppression, as this will reduce the chances of detecting the implant in the subcutaneous fat, and by reducing the overall signal, impair detection if it lies in deep muscle.

## 3 Implant Removal

### 3.1 Supplies and Instruments

- Sterile gloves
- Betadine or chlorhexidine gluconate
- 3–5 cc—1% or 2% lidocaine with or without epinephrine
- Gauze sponges
- Scalpel—#11 blade is best
- 2 fine‐tipped forceps (arteries/mosquito forceps)—at least one should be curved
- Suture—3–0 Vicryl is preferred (alternately, can use plain gut; however, NO silk)
- Band‐aid or gauze for covering incision
- Tape/plaster
- Headlamp

### 3.2 Steps for Removal

- Counsel the patient about removal. Encourage her to tell you if she feels numbness or tingling in her arm or hand during the procedure. This could indicate that you are manipulating a peripheral nerve and should stop manipulation in that spot and try to approach the implant from a different angle.
- Ultrasound the patient, if you have not done so already, to mark the location of the tip that is closest to the skin surface—usually the distal tip.
- Position the patient to be lying flat on her back with her arm bent into a goalpost position.
- Prep the proposed removal site with betadine or chlorhexidine.
- Inject ∼1 cc of lidocaine at the proposed incision site—reserve the remainder to use if the patient needs additional anesthesia.
- Make a small incision—∼5 mm.

#### 3.2.1 . If your implant is above the fascia

Using your mosquito forceps, bluntly dissect to the implant, grasp, and remove. In obese patients, the “blind” Mansour‐Walling technique with vasectomy forceps works well.

#### 3.2.2 If your implant is below the fascia

1. Using your mosquito forceps, bluntly dissect to the level of the fascia.
2. Grasp the fascia with your forceps and sharply incise, extend your fascial incision.
3. Using blunt dissection, grasp the implant and remove.
4. Visually inspect the implant. Measure and ensure it is the correct length and that no pieces have been left behind.
5. Close your incision in one or two layers if large enough to need stitches, and instruct patient to return for removal in 1 week or sooner if they are bothering her.
6. Cover with bandage.
7. Instruct patient to return for any signs of infection—expanding redness, progressive pain, purulent discharge, and fevers.

### 3.3 Troubleshooting

- If you cannot localize the implant—extend your incision (start with 1–2 cm, if you still cannot localize, extend incision up to 4 cm).
- If you feel like your incision is big enough but still cannot localize the implant, ensure that all of your layers are extended to the same size as your skin incision.
- If you have good visualization but still cannot localize the implant—use your ultrasound intra‐procedurally with a sterile probe cover.

## 4 Conclusion

This protocol provides a comprehensive stepwise approach for localization and removal of non‐palpable contraceptive implants. In general, most non‐palpable contraceptive implants can be localized with ultrasound using the “compression” and “no compression” sonographic techniques. Hence, no attempt should be made to remove an implant until it can be localized with ultrasound. In rare cases, the implant might still not be localized on ultrasound and may require additional imaging with x‐ray and/or MRI. Although deep subcutaneous implant insertions can be localized intraoperatively and removed through an incision made over the nearest end of the implant (distal or proximal), the secret for easy removal of sub‐facial insertions is to open up the fascia and directly visualize the implant below the facial plane before making an attempt to grasp and remove. This visualization almost always requires a larger incision (2 cm or greater). These steps should allow for successful removal of most deep or non‐palpable implants and can be adopted for practice in low‐middle income countries. This protocol may serve as a useful resource in broader clinical guidelines and training programs. Future comparative studies, including utilization of technological innovation in implant design (e.g., bio‐trackable implants) to prevent non‐palpability issues, could further refine its efficacy and adaptability.

## Author Contributions

Jaclyn M. Grentzer and Abraham Fessehaye Sium developed the concept and design of the protocol. Abraham Fessehaye Sium, Amani Nureddin Abdu, Jaclyn M. Grentzer, Sarah Prager, and Matthew F. Reeves contributed to the manuscript write‐up. Abraham Fessehaye Sium, Sarah Prager, and Matthew Reeves edited the final manuscript. All authors checked the manuscript for intellectual contents. The final manuscript is approved for submission by all authors.

## Disclosure

Abraham Fessehaye Sium is an editorial board member at Public Health Challenges, and he was excluded from the editorial decision‐making for this article.

## Conflicts of Interest

The authors declare no conflicts of interest.

## Data Availability

All data generated or analyzed during this study are included in this published article and its Supporting Information files.
